# Enhanced macroboring and depressed calcification drive net dissolution at high-CO_2_ coral reefs

**DOI:** 10.1098/rspb.2016.1742

**Published:** 2016-11-16

**Authors:** Ian C. Enochs, Derek P. Manzello, Graham Kolodziej, Sam H. C. Noonan, Lauren Valentino, Katharina E. Fabricius

**Affiliations:** 1 Cooperative Institute for Marine and Atmospheric Studies, Rosenstiel School of Marine and Atmospheric Science, University of Miami, 4600 Rickenbacker Cswy., Miami, FL 33149, USA; 2 Atlantic Oceanographic and Meteorological Laboratories (AOML), NOAA, 4301 Rickenbacker Cswy., Miami, FL 33149, USA; 3 Australian Institute of Marine Science, PMB 3, Townsville, Queensland 4810, Australia

**Keywords:** ocean acidification, coral reef, calcification, bioerosion, micro-CT, dissolution

## Abstract

Ocean acidification (OA) impacts the physiology of diverse marine taxa; among them corals that create complex reef framework structures. Biological processes operating on coral reef frameworks remain largely unknown from naturally high-carbon-dioxide (CO_2_) ecosystems. For the first time, we independently quantified the response of multiple functional groups instrumental in the construction and erosion of these frameworks (accretion, macroboring, microboring, and grazing) along natural OA gradients. We deployed blocks of dead coral skeleton for roughly 2 years at two reefs in Papua New Guinea, each experiencing volcanically enriched CO_2_, and employed high-resolution micro-computed tomography (micro-CT) to create three-dimensional models of changing skeletal structure. OA conditions were correlated with decreased calcification and increased macroboring, primarily by annelids, representing a group of bioeroders not previously known to respond to OA. Incubation of these blocks, using the alkalinity anomaly methodology, revealed a switch from net calcification to net dissolution at a pH of roughly 7.8, within Intergovernmental Panel on Climate Change's (IPCC) predictions for global ocean waters by the end of the century. Together these data represent the first comprehensive experimental study of bioerosion and calcification from a naturally high-CO_2_ reef ecosystem, where the processes of accelerated erosion and depressed calcification have combined to alter the permanence of this essential framework habitat.

## 1. Introduction

As ecosystem engineers, corals and coralline algae are responsible for the construction of habitat essential to the high biodiversity present on healthy reef ecosystems today [1,2]. Similar to forests and ubiquitous decomposers, on coral reefs bioeroding taxa break down and dissolve calcium carbonate skeletons [3]. It is this balance between positive (calcification) and negative (bioerosion) processes that is responsible for the formation and persistence of coral reef framework habitats. The rising partial pressure of carbon dioxide (CO_2_) and a concomitant decline in seawater pH (ocean acidification, OA) is predicted to favour net habitat loss, with widespread implications for reef ecosystem structure and function [4].

Numerous laboratory experiments [5,6] and several field studies [7–10] have demonstrated a link between OA and the depressed calcification of corals and algae, though different interspecific susceptibilities to OA stress can complicate ecosystem responses. Similarly, in controlled aquaria OA accelerates the chemical dissolution of coral skeletons by clionaid sponges [11,12] and well-illuminated microscopic chlorophytes [13,14]. Data from the field support this and there is a correlation between OA conditions and the prevalence of bivalves in the skeletons of living *Porites* corals [15–17], as well as fine-scale differences in pH and net community calcification [18]. Numerous other mechanisms of bioerosion (e.g. scraping and rasping) and bioeroding species (e.g. fishes, urchins, annelids, cirripedes) remain uninvestigated and it is unclear how they are responding to OA.

In order to understand the relative contribution of these diverse taxa, it is helpful to partition them into functional groups [3] that may then be combined within the context of reef framework dynamics [19,20]. Accordingly, calcifiers accrete calcium carbonate (CaCO_3_) materials to reef surfaces and include scleractinian corals, crustose coralline algae (CCA), sessile molluscs, as well as other taxa which are involved in forming more ephemeral structures, usually of little impact on framework construction (e.g. *Halimeda*, gastropods). Microborers, which include a diverse multi-phyletic consortium of flora and fauna, create boreholes in reef carbonates less than 100 µm in diameter. Animals forming larger cavities and tunnels are considered macroborers, and include clionaid sponges, annelids, bivalve molluscs, and crustaceans. Finally, grazing fauna, primarily fishes and urchins, remove carbonates from the surface of reef frameworks, often while feeding on benthic algae.

Differential responses to OA, coupled with a diversity of interrelated functional groups operating on reef habitat structure, give rise to the potential for complex and unforeseen outcomes. Bioeroding and calcifying taxa may interact through competition, facilitation, and predation, obscuring or exaggerating direct organismal responses to OA. For example, benthic algae, which may be enhanced by OA, can competitively restrict the growth of coral [21] and may encourage grazing by fishes and urchins. These grazers can remove large pieces of substrate from the surface, taking microborers, newly settling macroborer larvae, and exposed macroborers (e.g. sponge papillae) along with the coral rock, thereby restricting the proliferation of these groups [22–25]. Boring taxa weaken the substrate in which they dwell [26,27] and can enhance grazing rates [28], or even encourage substrate fracture and destruction by fishes foraging for invertebrates [29]. While these interactions are poorly studied, it is likely that the net result of OA on reef framework permanence will be more complex than the simple additive results of increased bioerosion and reduced calcification.

Analysis of this complex community is not feasible in a closed laboratory setting, where the absence of newly recruiting plankton and limited biodiversity preclude the natural processes of settlement, competition, and succession. Additionally, while experiments are generally run from weeks to months, the establishment of mature bioeroding and calcifying communities can take years [24]. Naturally high-CO_2_ systems, such as those due to upwelling [30], biological activity [31], groundwater intrusion [32], and volcanic venting [15] provide a means of examining complex real-world responses among communities that have existed in OA conditions for periods of time in excess of a decade.

The goal of this study was to simultaneously investigate the net impact of all functional groups responsible for reef framework persistence across a gradient of OA. We used a novel micro computed tomography (micro-CT) approach to independently quantify calcifiers, macroborers, microborers, and grazers influencing dead coral skeletons deployed at two volcanic CO_2_ vents in Papua New Guinea (PNG). This represents the first time bioerosion has been experimentally examined in dead reef framework material in a naturally high-CO_2_ coral reef and the first time that the influences of OA have been investigated simultaneously on multiple habitat-altering functional groups.

## 2. Material and methods

### (a) Construction and deployment

Bioerosion accretion replicates (BARs) were created to independently quantify the net result of accretion, grazing, macroboring, and microboring. Clean and unbored cores of massive *Porites* sp. coral were collected from the Great Barrier Reef and sectioned into 2 × 1 × 5 cm pieces. Each piece was affixed to a grey polyvinyl chloride (PVC) base (2 × 0.6 × 8 cm) using All-Fix underwater epoxy.

**Figure 1.**
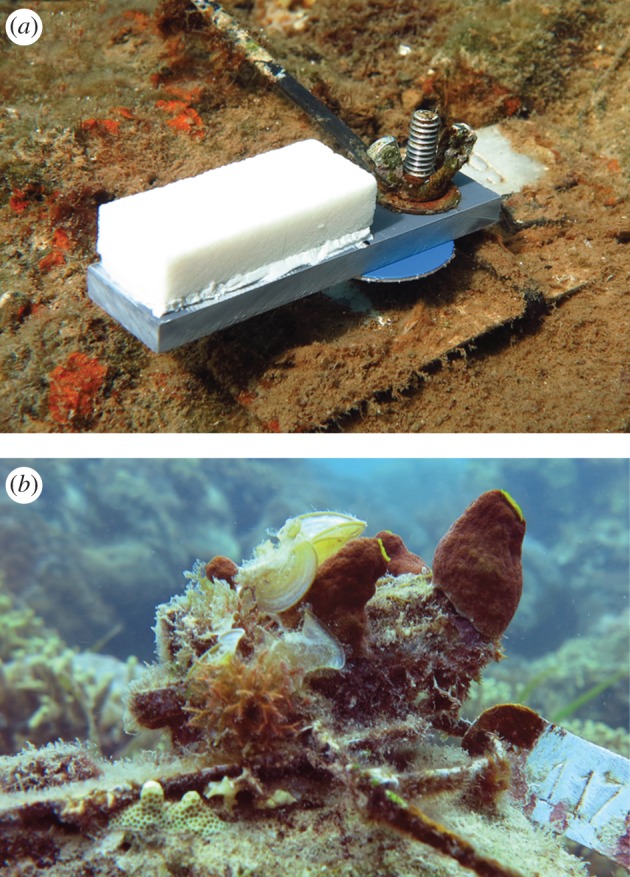
Bioerosion accretion replicates (BARs) used to quantify habitat persistence along OA gradients at the two sites. (*a*) Newly deployed uncolonized BAR affixed to the reef surface and (*b*) a different BAR after colonization by natural reef flora and fauna. BARs are roughly 5 × 2 × 1 cm.

BARs were deployed at sites surrounding volcanically acidified coral reefs at two islands (Dobu Island, and Illi-Illi Bwa Bwa near Upa-Upasina, Normandy Island) in Milne Bay Province, PNG (electronic supplementary material, figure S1). Description of these sites can be found in Fabricius *et al.* [15]. A total of 70 BARs were placed across the CO_2_ gradients at both reefs, spanning acidified to present-day control water chemistry. Each BAR was affixed to the substrate (approx. 3 m depth) using a single bolt and stable base pinned into bare coral rock (figure 1). Seawater samples were collected at each BAR site during four, two-week trips over the duration of the BARs' deployment (*n* = 5–23 per BAR location, 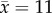 [8]), and pH (total scale) was measured using a high-accuracy glass electrode (InLab Expert Pro pH electrode, SG78 pH/temperature meter, Metler Toledo). Additional seawater samples were taken less frequently (*n* = 2–18 per site, 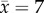) for analysis of total alkalinity (*A*_T_, 855 Titrosampler, Metrohm) and dissolved inorganic carbon (DIC, Vindta 3C, Marianda), which were used to solve the carbonate system (Seacarb v. 2.4.8, https://cran.r-project.org/web/packages/seacarb/index.html) and to confirm that pH was successfully describing the vent gradient. After 658–666 days (depending on the site), BARs were collected by divers. BARs were then incubated, as described below, to measure photosynthesis, respiration, calcification, and dissolution.

### (b) Incubations

After collection, the undersides and edges of the PVC base of the BARs were cleaned, without touching the epiphytes on the blocks and while keeping the blocks submerged in seawater at all times. They were then placed separately into custom-made, stirred clear acrylic incubation chambers (volume: 0.640 l; [9]), filled with seawater ranging from pH 8.1 to 7.6, manipulated via the addition of CO_2_-enriched seawater from the seep sites. Each BAR was incubated at a level of pH approximating the nearest 0.1 pH unit from its field deployment site. The chambers were placed on submersible stirring units in lots of eight in black bins with flow-through seawater for temperature control (29.0°C ± 0.46 s.d.). Each stirring unit contained a submersible motor and pulleys that created continuous water movement in each chamber via magnetic stir bars (35 mm bar at 200 r.p.m.). To determine net photosynthesis and light calcification, blocks were first incubated under illumination for approximately 80 min (AI Sol White LED Modules, Clear choice, Los Angeles, USA, set to 180 µmol photons m^−2^ s^−1^). At the end of the runs, the chambers were opened, and their Oxygen (O_2_) concentration determined with a hand-held dissolved oxygen meter (HQ30d, equipped with LDO101 IntelliCAL oxygen probe, Hach, USA). A subsample of seawater (250 ml) from each chamber was preserved in a sealed polycarbonate bottle with mercury chloride (HgCl_2_) for later determination of *A*_T_. To determine respiration and dark calcification, blocks were acclimatized to the dark for 0.5 h, and again transferred into the chambers newly filled with fresh seawater at their respective pH level. The chambers were closed and the bins were covered with black lids. After approximately 140 min incubation, the O_2_ concentrations from each chamber were again analysed and samples taken for *A*_T_. Initial water conditions were determined immediately prior to each round of incubations by obtaining one sample from bulk treatment water for analysis of *A*_T_ and O_2_. During both the light and dark incubations, two chambers per pH level were incubated without blocks, acting as control blanks. After incubations, the BARs were air dried for transport. Light and dark calcification rates were determined with the alkalinity anomaly technique [33]. Rates of net photosynthesis and dark respiration (µg O_2_ cm^−2^ min^−1^), and light and dark calcification (µmol CaCO_3_ cm^−2^ h^−1^) were calculated after subtracting the blank values. Net calcification was calculated assuming 11 h of daylight and 13 h of night time. All calcification, photosynthesis, and respiration values were normalized to the post-deployment surface area of each respective block as determined by micro-CT scanning.

### (c) CT analysis

**Figure 2.**
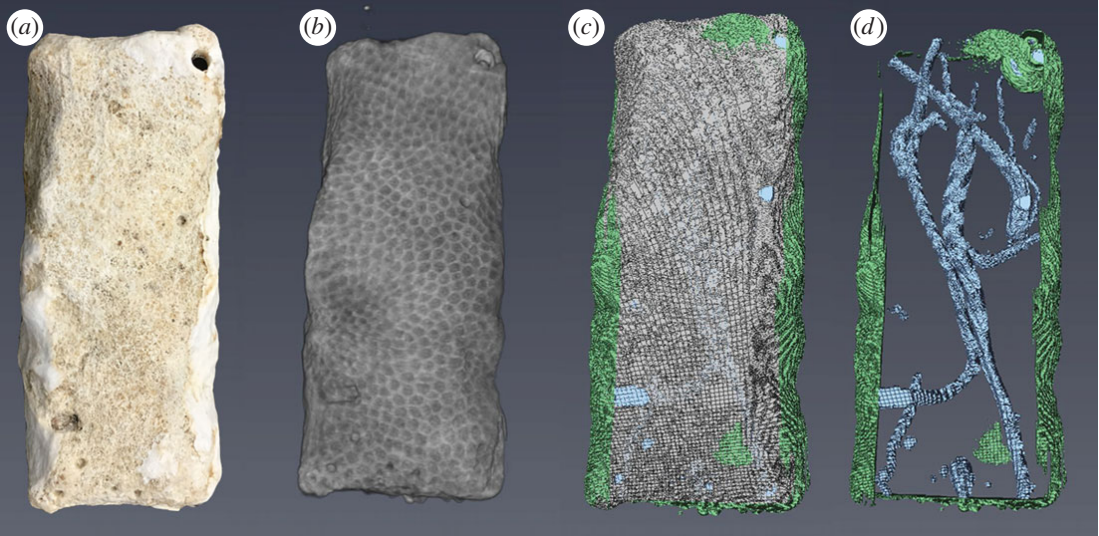
Analysis of bioerosion accretion replicates (BARs). (*a*) Photograph of a dry BAR; (*b*) three-dimensional reconstruction of micro-CT scan of the same BAR; (*c*) volumetric analysis showing original carbonate (grey), macroboring (blue), and crustose coralline algae (green); (*d*) view showing only macro-bioerosion and crustose coralline algae. BARs are roughly 5 × 2 × 1 cm.

BAR units were scanned before and after deployment using a micro-CT (Skyscan 1174, 50 kV, 800 µA). Initial and post-scans were run at 59.51 and 64.23 µm resolution, respectively. Initial scans were conducted on clean carbonate materials, dried for 24 h at 60°C. Organic matter was removed beforehand using a buffered sodium hydroxide (NaOH) solution and gentle abrasion. Scans were reconstructed into image stacks using NRecon (Bruker), a consistent ring artefact correction of 6, and a beam hardening correction of 40%. Custom-made coral aragonite density phantoms were analysed routinely using the same scan and reconstruction parameters, and were used to infer calcium carbonate density using linear regression of grams per cubic centimetre to X-ray attenuation (densitometry, see [30]). Isolation and volumetric quantification of accretion and bioerosion functional groups was conducted as follows using the Amira software package (FEI, figure 2).

1. Accretion by calcifiers was calculated as the volume of non-original carbonate material on the surface of the coral blocks.
2. Macroboring was calculated as the volume of void space within the remaining coral block.
3. Microboring was calculated as the difference in the density (pre- versus post-scan) of the non-bored, non-grazed BAR multiplied by the final volume of that area. It is noted that this number may also include changes in density due to abiotic dissolution/precipitation. Further, as the resolution of the micro-CT was set at 64.23 µm in the post-scan, it is possible that not all macroboring tunnels (more than 100 µm diameter) were resolved as this would effectively require thresholding of two pixels. These differences are instead detected in the microboring measurement. We therefore probably overestimated microboring rates and underestimated macroboring rates, though visual inspection of the scans suggests that this difference is minimal.
4. Grazing was calculated as the difference in external block volume (pre- versus post-scan), not inclusive of internal macroboring or external accretion.

Surface areas of pre- and post-scans were measured by creating three-dimensional isosurfaces following the exterior of each block using Amira and then by digitally removing the covered bottom surface and epoxy using the Leios 2 software package (EGS). Accretion and bioerosion functional groups were standardized to the initial surface area of each BAR.

### (d) Statistical analysis

Statistical analysis was conducted using RStudio [34] with ggplot2 [35]. Differences in functional groups were compared between the two reefs using non-parametric Wilcox tests for accretion, macroboring, and grazing, and using a *t*-test for microboring. Data from each reef were treated separately for subsequent analyses. Generalized linear models (GLMs) were run to examine the relationships between micro-CT quantified functional groups and carbonate chemistry in the field (mean pH), as well as incubation response metrics (photosynthesis, respiration, light calcification, dark calcification, 24 h calcification) and treatment pH. Two types of GLMs were employed based on the distribution of the data examined, either Gaussian (identity link function) or Gamma (log link function). Response variables analysed with Gamma GLMs which contained negative values were shifted before analysis and then transformed back to original before graphing. One block was excluded from incubation due to the presence of a large ascidian. One light and two dark *A*_T_ incubations were inconclusive and were eliminated from analysis, along with the accompanying three 24 h calcification estimates. An additional GLM was run on 24 h calcification data pooled from both reef sites in order to examine the point at which BARs switched from net accretion to net erosion.

## 3. Results

There were strong and consistent gradients in carbonate chemistry due to CO_2_ gas venting at the Dobu and Upa-Upasina sites (electronic supplementary material, figure S2). The Dobu reef spanned a larger gradient in mean pH (0.55 versus 0.34, Dobu versus Upa-Upasina, respectively). Seawater pH closely tracked DIC, pCO_2_, and aragonite saturation state (*Ω*_Arag_), demonstrating the ability of this more frequently measured parameter to describe the degree of OA incident on each BAR (electronic supplementary material, figure S2). Calculated values of *Ω*_Arag_ were consistently higher than 1.5, even at highly acidified sites, indicating that abiotic dissolution of this mineral phase was not highly favoured (electronic supplementary material, figure S2).

**Figure 3.**
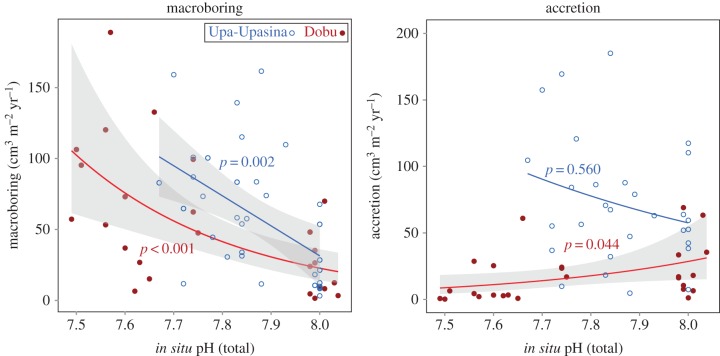
Relationship between pH and accretion, as well as pH and macroboring at two volcanically acidified coral reefs, Upa-Upasina (blue) and Dobu (red). *P*-values are associated with each GLM. Grey regions surrounding significant relationships are 95% CI. Five values from Upa-Upasina not shown in accretion panel in order to better visualize Dobu trend.

Diverse benthic communities colonized BARs during deployment (figure 1) and micro-CT analysis was successful at resolving and quantifying both calcifying and bioeroding functional groups (figure 2; electronic supplementary material, Video S1). The degree of macroboring within BARs was negatively related to pH at both vent sites (figure 3; electronic supplementary material, table S2). Borehole morphology within these samples indicated that the majority of this excavation was due to annelids, probably polychaetes (figure 2*d*). There was a significant positive relationship between the volume of newly accreted material and pH at the Dobu vent site, but not at Upa-Upasina (figure 3; electronic supplementary material, table S1). No significant relationship was found between pH and either grazing or microboring (electronic supplementary material, figure S3).

When functional group response data were pooled across pH treatments within each reef site, Dobu had significantly lower overall accretion (*W* = 104, *p* < 0.001) and higher grazing (*W* = 610, *p* = 0.002) than Upa-Upasina. Macroboring (*W* = 395, *p* = 0.338) and microboring were no different between sites (*t* = −5.245, d.f. = 53.229, *p* = 0.133).

**Figure 4.**
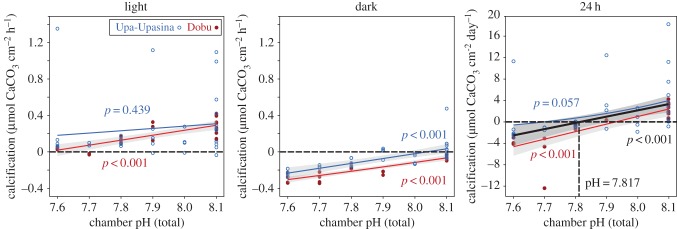
Relationship between chamber pH and net community calcification (light, dark, and 24 h) as determined by alkalinity anomaly incubations of bioerosion accretion replicates (BARs) deployed at two volcanically acidified coral reefs, Upa-Upasina (blue) and Dobu (red). *P*-values are associated with each GLM. Grey regions surrounding significant relationships are 95% CI. Horizontal dashed line shows no net accretion or dissolution. Vertical dashed line in 24 h panel highlights the pH at which BAR replicates switch from net accretion to net erosion, averaged over both sites.

Incubations revealed a significant positive relationship between light calcification and pH treatments for BARs deployed at Dobu, but not for those from Upa-Upasina (figure 4; electronic supplementary material, table S1). Dark calcification, however, was positively correlated with pH for BARs collected from both vent sites (figure 4; electronic supplementary material, table S1). Averaged across the two sites, 24 h averaged calcification switched from net positive to net dissolution at a pH of roughly 7.8. While the relationship between pH and 24 h calcification was significant among samples collected from Dobu, this was not true of those collected from Upa-Upasina (figure 4 and electronic supplementary material, table S1). No significant relationships between pH and photosynthesis were observed (electronic supplementary material, figure S4 and table S1). Respiration was observed to increase (greater oxygen consumption) with declining pH on the BARs from Dobu but not Upa-Upasina (electronic supplementary material, figure S4 and table S1).

## 4. Discussion

### (a) Relationship between ocean acidification and bioeroding functional groups

The strong significant relationship between pH and macroboring observed in the naturally acidified reefs in PNG is in line with previous studies of boring Porifera [11,12] and Chlorophyta [13,14] subjected to experimentally manipulated OA conditions in laboratory environments. To our knowledge, these data are the first measurements of elevated macroboring rates within dead reef carbonates in naturally high-pCO_2_ conditions and indicate that macroboring of reef frameworks will continue to accelerate due to OA.

This study also represents the first that we are aware of to demonstrate a relationship between the boring activity of annelids and OA. While information on the bioerosion rates of annelids is scarce, data from the Great Barrier Reef suggests that, on average, they remove more carbonate per unit area of dead reef framework than any other macroboring taxon, reaching rates as high as 1.788 kg m^−2^ yr^−1^ [36]. As such, evidence of their increased prevalence within substrates subjected to high-pCO_2_ water has strong ramifications for reef persistence under OA.

While the mechanisms of carbonate dissolution are not well known for all annelid taxa, similar to boring bivalves, they are likely to involve both chemical dissolution and mechanical abrasion [37]. To date, all bioeroding taxa known to respond to OA employ some form of chemical dissolution and those species strictly employing mechanical erosion, such as fishes and urchins, have not been found to accelerate erosion under OA conditions. This is corroborated by a lack of a significant relationship between external grazing and pH in this study (electronic supplementary material, figure S3).

While macroboring was higher at acidified sites and experimental studies support a direct relationship between OA and biologically mediated chemical dissolution of carbonates [11–14], we cannot conclusively state that this occurred herein. For example, previous studies have observed a higher prevalence of macroboring worms in damselfish territories, where large herbivores were excluded and microalgae was higher [23]. While we did not observe a significant relationship between pH and photosynthesis during BAR incubations (electronic supplementary material, figure S4), OA enhancement of algae *in situ* [38] may have encouraged annelid erosion near the vent sites. Targeted experiments are necessary to establish mechanisms, though indirect effects and species interactions will have strong implications for ecosystem structure under OA and are often underexplored [39].

With one exception [40], previous studies of bioerosion from naturally high-CO_2_ systems have focused on the prevalence of macroborers colonizing live *Porites* coral [15,16,17,32]. While these studies are highly informative, bioeroding communities within living coral skeletons are different than those occupying dead coral substrates, where the majority of coral reef bioerosion occurs [41]. Bioerosion within living corals is inherently complicated by species interactions [42], and it can be difficult to completely separate net dissolution from the influence of calcification, which is often strongly dependent on OA [6]. By contrast, dead coral blocks deployed in this study underwent the natural processes of colonization and succession that would be expected to occur after coral mortality. OA acceleration of macroboring within these substrates indicates the potential for a shift to a net erosive reef state and diminished reef habitat persistence following coral mortality.

In contrast with previous laboratory [13,14] and field studies [39], there was no significant relationship between microboring and seawater pH (electronic supplementary material, figure S3). This study is the first to analyse microboring within coral rock substrates, along a natural OA gradient. Species interactions could have influenced this relationship, making it more difficult to detect OA enhancement of bioerosion rates. For example, grazing, which was high at both sites (electronic supplementary material, figure S3), can remove material that had been infested by microboring communities, resulting in an underestimation of the latter [24,25]. Alternatively, external colonization of BAR surfaces could have inhibited microborer settlement [43] or material removed by macroborers could have decreased the power of our analysis to resolve the response of microborers. While these results suggest that OA enhancement of microboring is less ecologically important than macroboring and grazing, it must be noted that the initial colonization and dissolution of substrates by microborers may facilitate the subsequent colonization of macroborers and may enhance grazing of fishes, which obtain nutrients from substrates replete with endolithic algae [44,45]. OA enhancement of early-stage microborer communities may therefore indirectly accelerate macroborer and grazing communities through the modification of substrate and the availability of food.

### (b) Relationship between ocean acidification and calcifiers

Accretion to BAR units was negatively correlated with OA at the Dobu reef site. This newly calcified material was deposited by diverse taxa (polychaetes, molluscs, scleractinians, and CCA) precipitating multiple carbonate mineral phases, all of which will probably be impacted by OA [46,47]. The fact that a significant relationship between OA and accretion was only observed at one of the two study sites is interesting and may be due to the more extreme pH gradient at Dobu, which could have increased the ability to detect an OA signal. Additionally, Dobu had significantly higher grazing and lower overall accretion than Upa-Upasina, which may have contributed to the degree to which pH influenced new calcification (figure 3; electronic supplementary material, figure S3).

One of the dominant encrusting taxa, CCA, is well known to be negatively influenced by OA [5,38], and several researchers have documented depressed coverage of CCA using settlement tiles deployed along natural CO_2_ gradients [8,39,48]. In the light of these studies, the lack of a significant relationship between accretion and Upa-Upasina may, at first glance, appear incongruous. The methodologies employed in these studies, however, differ from those used herein in two fundamental ways, which should be considered when comparing results. First, per cent cover was previously used as a metric to quantify just the CCA community, rather than the volumetric quantification of the entire calcifying community reported here. As such, in this study the presence of other large calcifying taxa (e.g. molluscs and scleractinians), rather than thin CCA crusts, may have increased variability and obscured the trends previous authors reported from data based on per cent cover of a single taxon. Secondly, while evidence of grazing was apparent in the aforementioned studies, settlement tiles were constructed from non-carbonate materials (PVC and volcanic rock) that were likely more resistant to erosion during abrasion by fishes and echinoids. While the potential for greater substrate removal by grazing may complicate accretion data, this process would occur to the same degree on natural coral skeletons and frameworks, and should therefore be considered when predicting reef responses to OA.

### (c) Calcification and dissolution

Correlation between net calcification and pH treatments from the incubation experiments corroborated data from the micro-CT analysis and supports the hypothesis that OA will impair net community calcification. No significant relationships were observed between OA and photosynthesis (electronic supplementary material, figure S4 and table S1), which is in contrast with some studies which have found photosynthetic enhancement of algae in high-pCO_2_ conditions [38]. These responses can, however, be species dependent and are not always clear [38]. Additionally, high grazing rates may have reduced algae biomass to a level where detection of these patterns was limited. Respiration, by contrast, was positively correlated with pH at Dobu (electronic supplementary material, figure S4 and table S1), which is consistent with previous laboratory studies [49] and may be reflective of higher biomass of epibiota and macroborers, or stress.

While calcification data are in agreement with previous alkalinity anomaly experiments, it should be noted that they are not necessarily directly comparable [50–52]. The chemical responses quantified in this study were from communities that were allowed to establish and undergo succession under treatment conditions, rather than mature assemblages introduced into future OA scenarios. As noted previously, early life-history stages can be especially sensitive to OA stress, potentially resulting in reduced recruitment success and altered community composition [53]. Similarly, differential alteration of functional groups can disrupt competitive balances [21] and impact succession and community development. In these ways, net calcification under real OA conditions will reflect changes in community composition as well as directly altered physiology. Unlike this study, experimental OA conditions briefly applied to communities acclimatized to present-day carbonate chemistry may therefore overestimate calcification under future OA levels.

Incubation data in this and previous studies are chemical in nature and are not inclusive of the numerically dominant influence of physical grazing (electronic supplementary material, figure S3) and the high proportion of mechanical erosion that can occur with some macroboring taxa (e.g. sponges, [54]). It is likely that true net erosion of these BARs would be reached at even milder OA treatment conditions (higher pH) than the 7.8 mean chamber pH measured during the incubations (figure 4) because physical loss of reef structure is the result of both mechanical bioerosion and chemical dissolution.

### (d) Regional comparisons and wider implications

A similar volcanically acidified system at Maug Island, 'Commonwealth of the Northern Mariana Islands (CNMI) showed a complete loss of carbonate frameworks at a mean pH of 7.9 [7] and at a reef off Iwotorishima, Japan, reef framework habitats were replaced by soft-coral dominated systems at a mean pH 7.8 [55]. In the Galapagos, El Niño related warming has led to widespread coral mortality. Reef frameworks have since eroded away where upwelling results in nutrient-rich waters with a pH lower than 8.0, whereas frameworks and corals still persist in the higher latitudes, less influenced by upwelling [30]. In contrast with the aforementioned reef regions, reef frameworks in PNG demonstrate a degree of OA resilience and are present at a mean pH of 7.8 due to space monopolization by robust massive *Porites* corals. In these low-pH areas, however, CCA cover is significantly lower, coral rubble is sparse (unpublished data), and coral communities are low in diversity and structural complexity [15]. In Palau, biological activity in sheltered lagoons with high residence times drives seawater pH to as low as 7.84 [31]. Despite these extreme OA conditions, reefs maintain high coral cover, diversity, and calcification, though macroboring of living corals is accelerated [17,31]. The different resilience/susceptibility of these reef systems potentially indicates a degree of ecosystem plasticity. Presently, direct comparison of these naturally high-CO_2_ ecosystems is limited due to unquantified variation in extraneous environmental factors and mean pH may not be the best metric for comparing OA conditions across sites. Differences in CO_2_ dynamics and disturbance histories (e.g. bleaching), as well as concurrent physical (e.g. water flow), chemical (e.g. nutrients), and ecological factors (e.g. competition) have the potential to exacerbate or ameliorate biological responses to OA, or even impact the chemical influences of OA itself [56]. These factors are in need of further characterization and should be considered when comparing naturally acidified sites.

Together, the functional group responses of macroborers and calcifiers, coupled with the net community responses quantified in the incubations, represent empirical evidence that OA will favour net habitat loss through a two-front assault on carbonate persistence; namely an acceleration of dissolution and a decrease in calcification. This will lead to a decrease in architectural complexity and a loss in essential habitat for diverse biota [4,57]. Additional factors impacting coral reefs at regional (e.g. nutrients, overfishing) and global scales (warming) may further push reefs from accretion to erosion, and multiple-stressor experiments are needed within naturally high-CO_2_ environments to better understand the confluence of these factors.

## Supplementary Material

Supplementary Figures

## Supplementary Material

Supplementary Tables

## Data accessibility

Data are available through NOAA's National Centers for Environmental Information (NCEI) and can be accessed at http://accession.nodc.noaa.gov/0156692.
