# Supplementary material for: Enhanced macroboring and depressed calcification drive net dissolution at high-CO2 coral reefs
Source: Proc Biol Sci. 2016 Nov 16;283(1842):20161742. doi: 10.1098/rspb.2016.1742 (PMC5124095; doi:10.1098/rspb.2016.1742)
Supplement: Supplementary Figures [file rspb20161742supp1.docx]

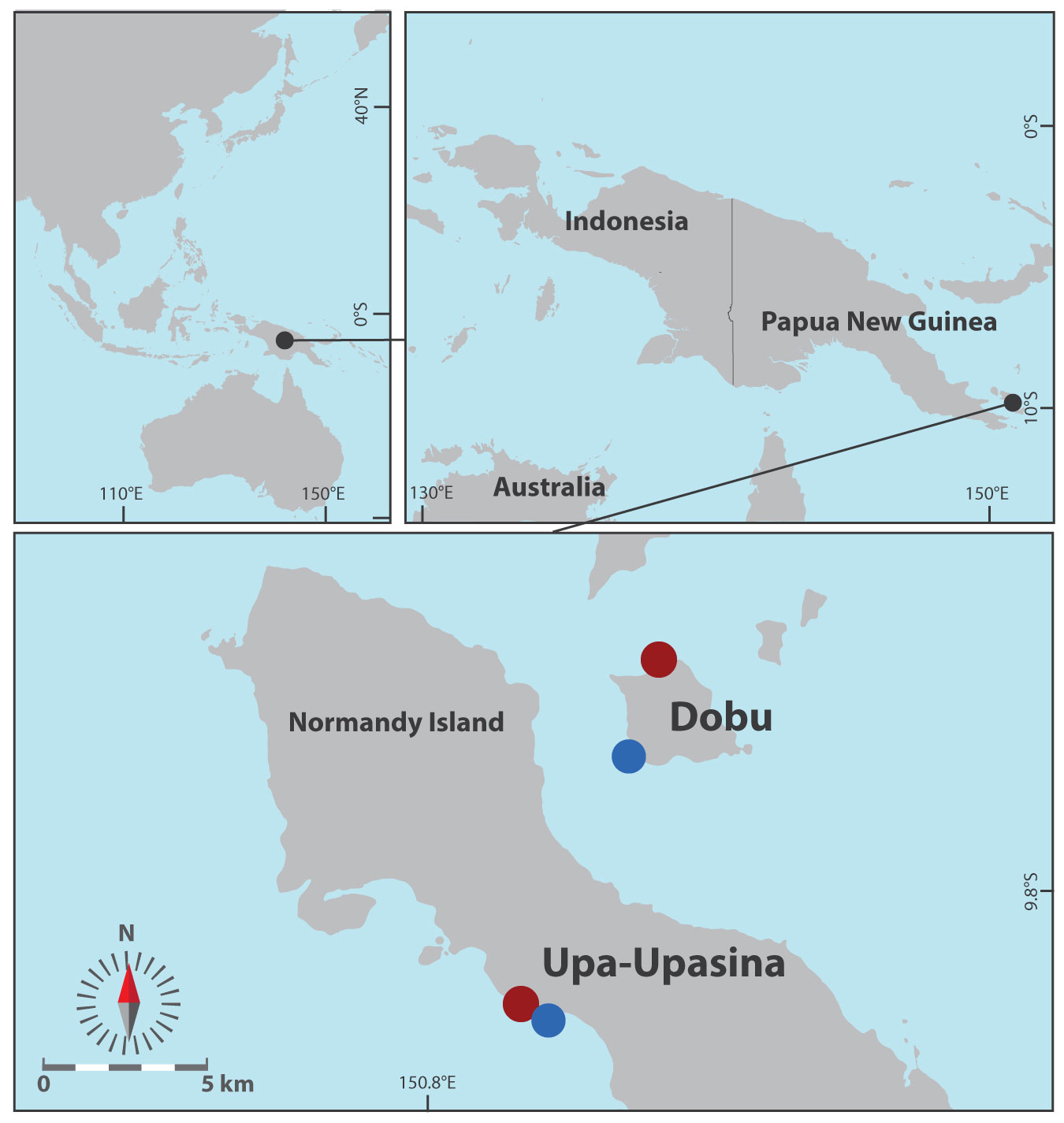


**Figure S1.** Map showing the location of the Dobu and Upa-Upasina sites. Red and blue dots indicate the locations of vent and control sites, respectively.

**
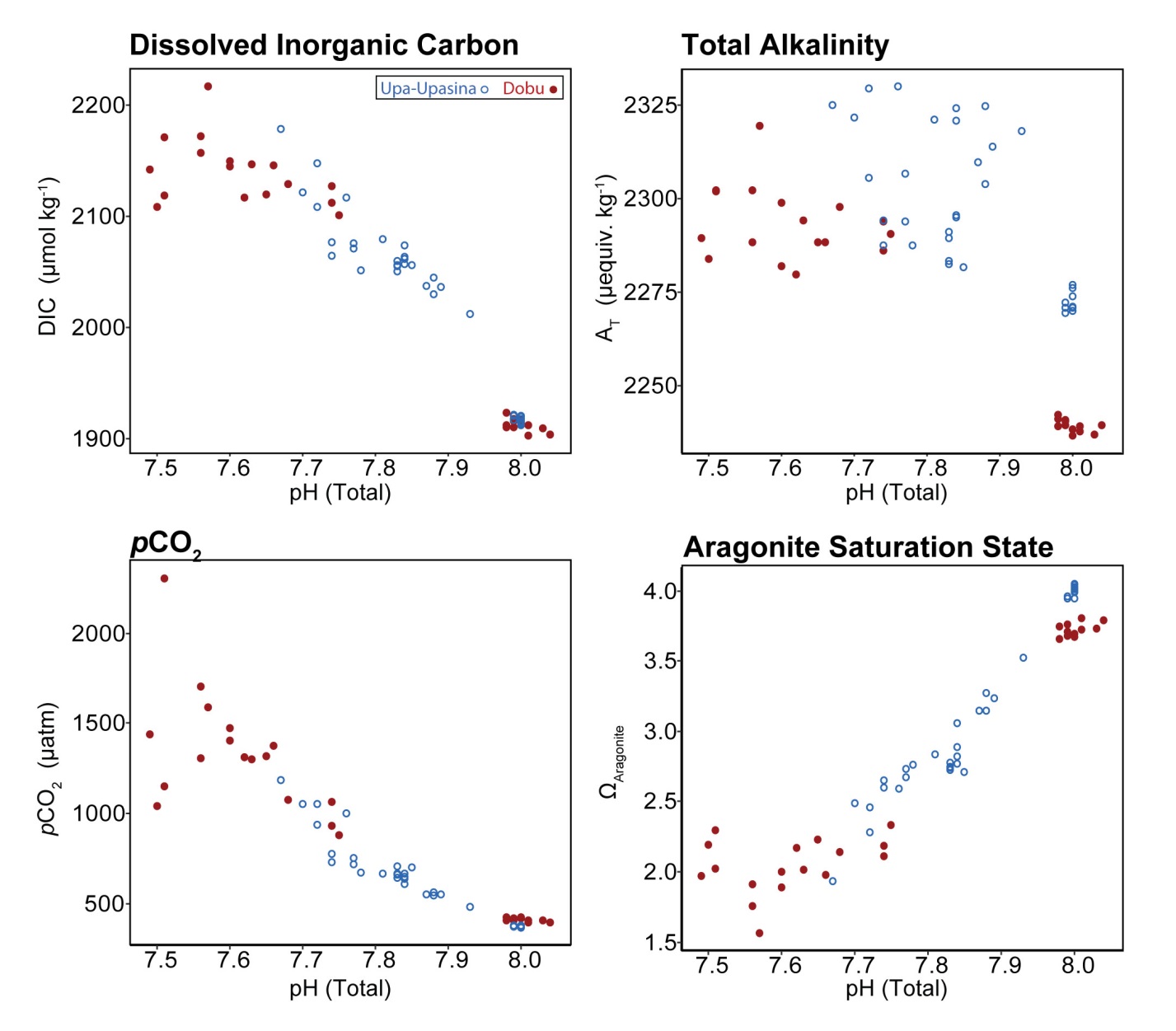
**

**Figure S2.** Relationship between carbonate chemistry parameters and seawater pH at BAR deployment sites at Dobu (red) and Upa-Upasina (blue).

**
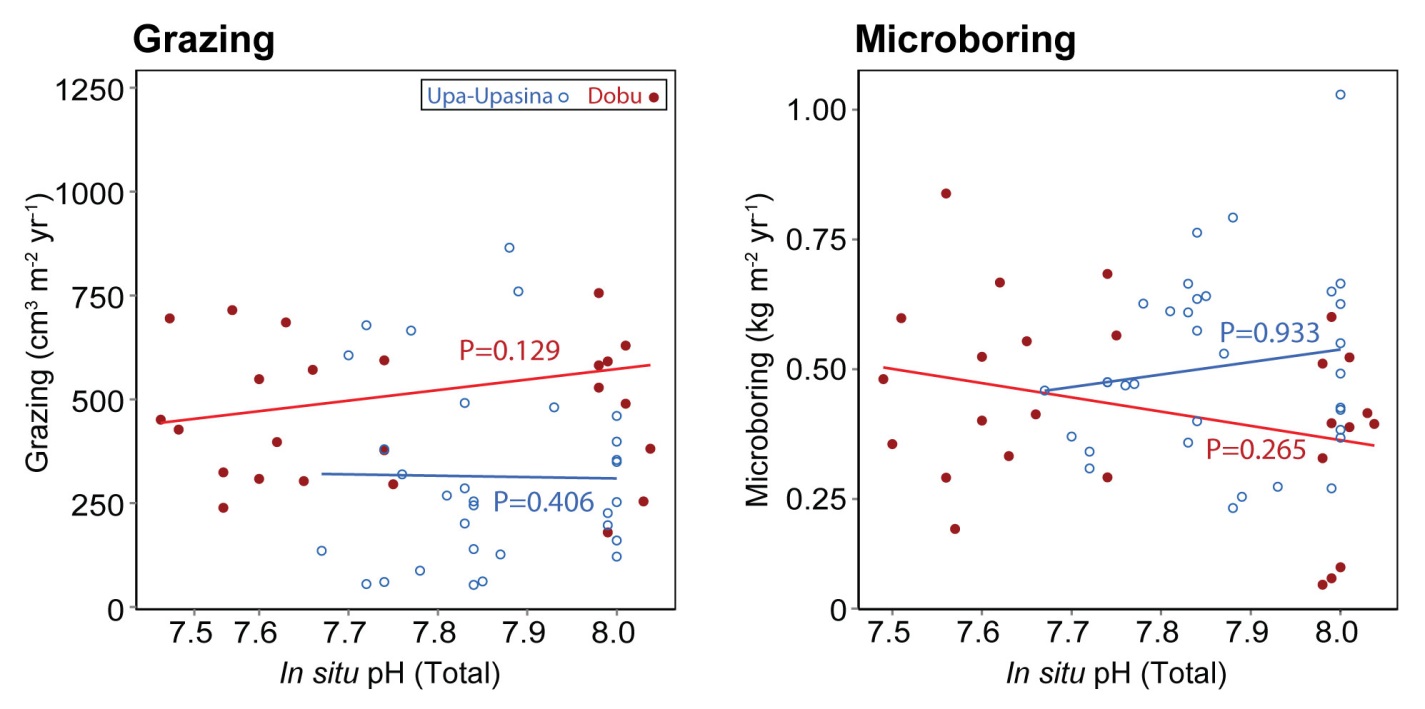
**

**Figure S3.** Relationship between pH and grazing, as well as pH and microboring at two volcanically acidified coral reefs, Upa-Upasina (blue) and Dobu (red). P values reveal no significance with associated GLM’s. Grazing presented as volume of material removed. Microboring presented as mass removed as measured by densitometry.

**
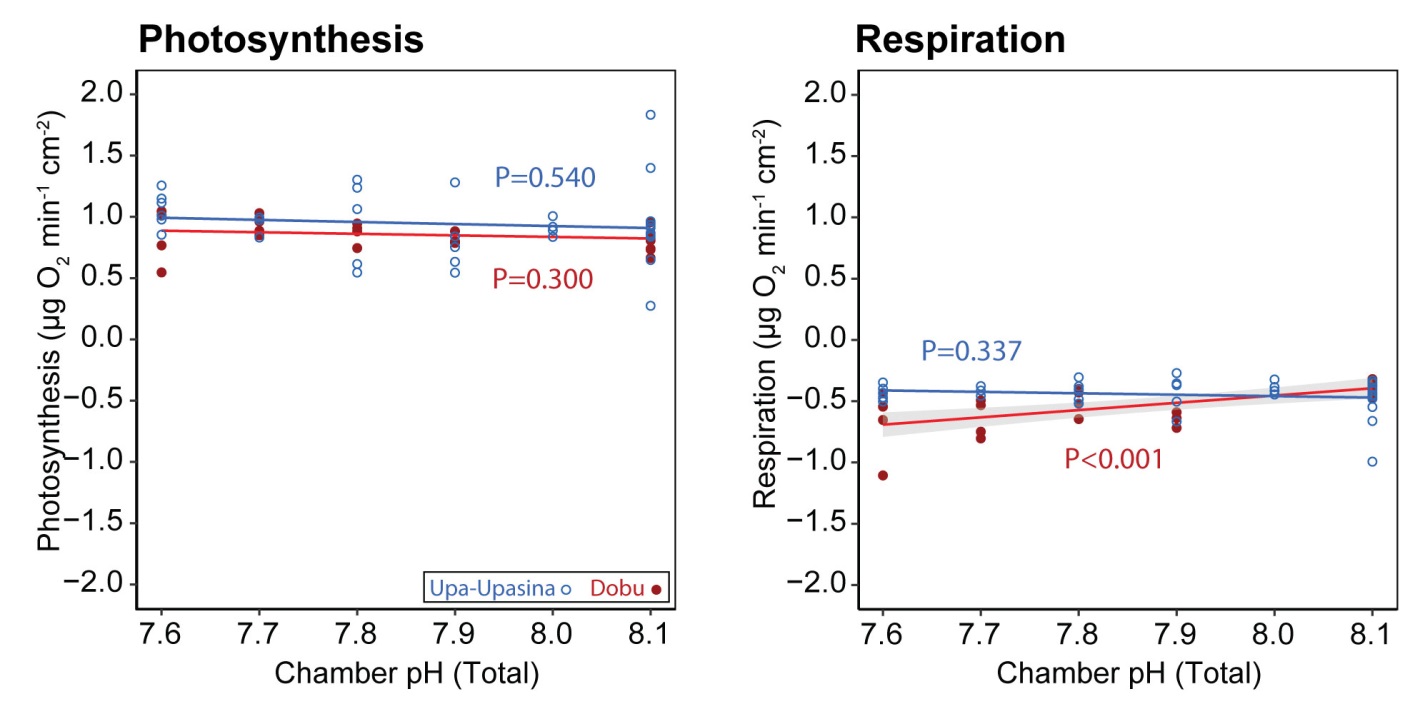
**

**Figure S4.** Relationship between chamber pH and photosynthesis, as well as chamber pH and respiration, as determined by sealed incubation of bioerosion accretion replicates (BARs) deployed at two volcanically acidified coral reefs, Upa-Upasina (blue) and Dobu (red). P values associated with GLM’s, grey regions surrounding significant relationships are 95%CI.

**Video S1.** Three-dimensional volume rendering of bioerosion accretion replicate (BAR), showing original carbonate block in grey, new accretion in green, and annelid boreholes in red.
