# Supplementary material for: Enhanced macroboring and depressed calcification drive net dissolution at high-CO2 coral reefs
Source: Proc Biol Sci. 2016 Nov 16;283(1842):20161742. doi: 10.1098/rspb.2016.1742 (PMC5124095; doi:10.1098/rspb.2016.1742)
Supplement: Supplementary Tables [file rspb20161742supp2.docx]

**Table S1.** Model statistics used for the analysis of the relationship between pH and BAR incubations. dF, degrees of freedom; AIC, Akaike Information Criterion; SE, standard error; t, t value; P, P value. Values marked with * are considered significant.

| **Parameter** | **Location** | **Model** | **df** | **AIC** | **SE** | **t** | **P** |
| --- | --- | --- | --- | --- | --- | --- | --- |
| **Photosynthesis** | Dobu | Gaussian | 23 | -32.371 | 0.120 | -1.061 | 0.300 |
|  | Upa-Upasina | Gamma | 32 | 16.501 | 0.285 | -0.620 | 0.540 |
| **Respiration** | Dobu | Gaussian | 23 | -22.101 | 0.147 | 4.031 | <0.001* |
|  | Upa-Upasina | Gaussian | 32 | -38.192 | 0.120 | -0.974 | 0.337 |
| **Light Calcification** | Dobu | Gaussian | 22 | -9.685 | 0.190 | 3.739 | 0.001* |
|  | Upa-Upasina | Gamma | 32 | 23.478 | 0.145 | 0.783 | 0.439 |
| **Dark Calcification** | Dobu | Gamma | 22 | -71.973 | 0.028 | 9.450 | <0.001* |
|  | Upa-Upasina | Gaussian | 31 | -54.403 | 0.092 | 5.812 | <0.001* |
| **24-Hr Calcification** | Dobu | Gaussian | 21 | 108.210 | 2.438 | 5.758 | <0.001* |
|  | Upa-Upasina | Gamma | 31 | 169.130 | 1.071 | 1.974 | 0.057 |
|  | Combined | Gaussian | 54 | 319.250 | 2.784 | 4.184 | <0.001* |

**Table S2.** Model statistics used for the analysis of the relationship between pH and functional groups impacting BAR volume (accretion, macroboring, grazing) and density (microboring). dF, degrees of freedom; AIC, Akaike Information Criterion; SE, standard error; t, t value; P, P value. Values marked with * are considered significant.

| **Group** | **Location** | **Model** | **df** | **AIC** | **SE** | **t** | **P** |
| --- | --- | --- | --- | --- | --- | --- | --- |
| **Accretion** | Dobu | Gamma | 24 | 204.58 | 1.115 | 2.124 | 0.044* |
|  | Upa-Upasina | Gamma | 32 | 392.77 | 1.672 | 0.590 | 0.560 |
| **Macroboring** | Dobu | Gamma | 24 | 253.62 | 0.786 | -3.842 | <0.001* |
|  | Upa-Upasina | Gaussian | 32 | 349.47 | 63.58 | -3.331 | 0.002* |
| **Microboring** | Dobu | Gaussian | 24 | -11.58 | 0.175 | -1.571 | 0.265 |
|  | Upa-Upasina | Gaussian | 32 | -18.11 | 0.286 | 0.842 | 0.933 |
| **Grazing** | Dobu | Gaussian | 24 | 360.22 | 222.3 | 1.142 | 0.129 |
|  | Upa-Upasina | Gamma | 29 | 417.56 | 1.224 | -0.085 | 0.406 |
